# 基于公共数据库探讨DDB1在肺腺癌中的表达和作用

**DOI:** 10.3779/j.issn.1009-3419.2025.102.12

**Published:** 2025-04-20

**Authors:** Xinkai ZOU, Ziyi HE, Yanfei ZHANG, Yi HU, Xiaomin WANG, Zhongjie WU

**Affiliations:** ^1^314000 嘉兴，浙江中医药大学嘉兴大学联培基地（邹昕锴，何子轶）; ^1^Jiaxing University Master Degree Cultivation Base, Zhejiang Chinese Medical University, Jiaxing 314000, China; ^2^314000 嘉兴，嘉兴市第一医院心胸外科（张雁飞，胡奕，吴中杰）; ^2^Department of Cardiothoracic Surgery,The First Hospital of Jiaxing, Jiaxing 314000, China; ^3^314000 嘉兴，嘉兴大学医学院细胞生物学系（王晓敏）; ^3^Department of Cell Biology, College of Medicine, Jiaxing University, Jiaxing 314000, China

**Keywords:** 肺肿瘤, DDB1, 预后, 生物标志物, Lung neoplasms, DDB1, Prognosis, Biomarker

## Abstract

**背景与目的:**

肺腺癌（lung adenocarcinoma, LUAD）是非小细胞肺癌（non-small cell lung cancer, NSCLC）的主要亚型。损伤特异性DNA结合蛋白1（damage-specific DNA binding protein 1, DDB1）作为CUL4-DDB1泛素连接酶复合体的核心蛋白，参与调控DNA损伤修复、表观遗传修饰及细胞周期检查点激活等关键生物学过程。有报道DDB1通过DNA修复与RNA转录调控参与肿瘤进展，但其在LUAD中的表达及作用尚不明确。本研究旨在探讨DDB1在LUAD中的表达和作用。

**方法:**

利用UALCAN、Kaplan-Meier Plotter、GEPIA等数据库，对DDB1在LUAD中的表达及预后、临床病理特征等进行分析；通过GeneMANIA和Metascape构建相互作用网络及富集功能通路；结合TISIDB评估DDB1与免疫细胞浸润相关性，通过单细胞测序对细胞亚型的聚类结果和DDB1在不同免疫细胞亚群中的表达情况进行分析；最后，利用组织芯片进一步验证DDB1在LUAD中的表达及预后价值。

**结果:**

DDB1在LUAD组织中的表达显著高于正常组织（P<0.01），且高表达与肿瘤原发灶-淋巴结-转移（tumor-node-metastasis, TNM）分期较晚（P<0.001）、淋巴结转移（P<0.001）及预后不良（P<0.001）有关。功能富集显示DDB1参与DNA修复与RNA转录调控。TISIDB评估发现DDB1与免疫细胞的表达水平呈负相关，提示存在免疫微环境调控潜力。单细胞分析显示，DDB1主要在T细胞、肺泡巨噬细胞以及树突状细胞中表达。组织芯片证实DDB1高表达组总生存期更短（P<0.001），Cox多因素分析表明DDB1为预测LUAD预后的独立因子。

**结论:**

DDB1在LUAD中高表达，与患者后不良有关，并且与肿瘤免疫细胞浸润密切相关，通过DNA修复与RNA转录调控参与肿瘤发生发展。DDB1可作为LUAD潜在的预后标志物及治疗靶点。

## Abstract (en)

**Background and objective:**

Lung adenocarcinoma (LUAD) is the predominant subtype of non-small cell lung cancer (NSCLC). Damage-specific DNA binding protein 1 (DDB1), as a core protein of the CUL4-DDB1 ubiquitin ligase complex, is involved in the regulation of DNA damage repair, epigenetic modification, and cell cycle checkpoint activation. While the involvement of DDB1 in tumour progression through DNA repair and RNA transcriptional regulation has been reported, its expression and role in LUAD remain to be elucidated. This study aims to investigate the expression and role of DDB1 in LUAD.

**Methods:**

The expression, clinicopathological features and prognosis of DDB1 in LUAD were analysed using databases such as UALCAN, Kaplan-Meier Plotter and GEPIA; The interaction network and enriched functional pathways were constructed by GeneMANIA and Metascape; the correlation between DDB1 and immune cells by combining with TISIDB infiltration was evaluated, and the clustering results of cell subtypes and the expression of DDB1 in different immune cell subpopulations were analysed by single-cell sequencing; finally, tissue microarrays were used to further verify the expression and prognostic value of DDB1 in LUAD.

**Results:**

The mRNA and protein expression of DDB1 in LUAD tissues were significantly higher than those in normal tissues (P<0.01), and the high expression correlated with later clinical stage (P<0.001), lymph node metastasis (P<0.001) and poor prognosis (P<0.001). Functional enrichment showed that DDB1 was involved in DNA repair and RNA transcriptional regulation, and TISIDB evaluation revealed that DDB1 was negatively correlated with the expression level of immune cells, suggesting the potential regulation of the immune microenvironment. Single cell analysis showed that DDB1 was mainly expressed in T cells, alveolar macrophages and dendritic cells. Tissue microarrays confirmed that overall survival was shorter in the DDB1 high expression group (P<0.001), and Cox multifactorial analysis showed that DDB1 was an independent predictor of LUAD prognosis.

**Conclusion:**

DDB1 is highly expressed in LUAD, which is associated with poor prognosis, and is closely related to tumor immune cell infiltration, and is involved in tumourigenesis and development through DNA repair and RNA transcriptional regulation. DDB1 can be used as a potential prognostic marker and therapeutic target for LUAD.

肺癌是世界上最常见的恶性肿瘤之一^[1]^，主要分为小细胞肺癌（small cell lung cancer, SCLC）和非小细胞肺癌（non-small cell lung cancer, NSCLC）。在NSCLC的临床分型中，肺腺癌（lung adenocarcinoma, LUAD）是最具侵袭性的病理亚型。LUAD不仅具有高度异质化的生物学特性，其发生发展过程常源于腺体黏膜的恶性转化，并伴随复杂的基因变异网络，呈现出显著的突变负荷特征^[2]^。虽然精准医疗时代下的靶向疗法、免疫调控策略以及血管生成抑制方案已显著改善临床转归，但对于疾病晚期的患者群体，长期生存率仍难以突破15%^[3]^。研究^[4]^表明，肺癌的分子机制主要涉及原癌基因异常活化、抑癌通路失活以及DNA修复系统功能障碍等多重生物学过程。例如，携带表皮生长因子受体（epidermal growth factor receptor, EGFR）激活突变的NSCLC能用该受体的特异性酪氨酸激酶抑制剂（tyrosine kinase inhibitors, TKIs）治疗^[5]^。突变的Kirsten大鼠肉瘤病毒癌基因同源物（Kirsten rat sarcoma viral oncogene homolog, KRAS）激活了LUAD细胞中CD47的表达，导致巨噬细胞对癌细胞的吞噬作用减少^[6]^。在NSCLC，p53突变失活会导致对顺铂的耐药性^[7]^。目前临床实践中，针对性靶向药物被用于控制肺癌进展，然而耐药现象制约了治疗效果。为此，深入阐明其病理机制并发现潜在治疗靶标，成为突破现有治疗瓶颈的重要方向。

损伤特异性DNA结合蛋白1（damage-specific DNA binding protein 1, DDB1）作为CUL4-DDB1泛素连接酶复合体的核心衔接蛋白，在调控该复合体功能中扮演着枢纽角色。其独特的β-propeller结构域能够动态结合多种DDB1-CUL4相关因子（DDB1-CUL4 associated factors, DCAFs）底物受体，形成高度特异性的底物识别模块^[8]^。这种模块化设计使CUL4-DDB1复合体能够通过替换不同DCAFs具有广泛的底物选择性，进而参与调控DNA损伤修复、表观遗传修饰及细胞周期检查点激活等关键生物学过程^[9]^。

DDB1在多种肿瘤组织中异常高表达。DDB1在卵巢癌中的表达具有异质性，这表明它可作为卵巢癌生存率低的潜在生物标志物^[10]^。DDB1通过DNA损伤修复，在膀胱癌中发挥促癌因子的作用^[11]^。然而DDB1在LUAD的发生和发展中的作用尚未得到深入研究。本研究系统探讨DDB1在LUAD中的生物学作用，通过多组学方法验证其临床相关性，评估其作为新型诊疗标志物的可行性，并探索靶向干预的潜在途径。

## 1 资料与方法

### 1.1 临床组织

研究使用的人肺组织芯片（HLugA180Su11）系上海芯超生物科技有限公司研发并提供。该芯片包含89例肺癌肿瘤组织以及90例癌旁组织，所有样本均具备完整临床数据。伦理审批经上海芯超生物科技有限公司伦理委员会审核通过（批准号：SHYJS-CP-2206001）。研究涉及的部分临床信息由商业来源提供。

### 1.2 UALCAN分析

UALCAN是癌症基因组学分析平台，其数据源自癌症基因组图谱计划（The Cancer Genome Atlas, TCGA）数据库。该平台可检测基因在肿瘤组织与健康样本中的表达差异，同时支持将基因表达水平与患者临床特征进行比对分析。具体而言，通过输入DDB1基因，选择LUAD癌症类型，执行"Expression Analysis"模块。系统自动生成基因表达箱线图，并提供P值及Log-rank检验结果。研究聚焦DDB1 mRNA及蛋白表达差异，重点分析其在肿瘤与正常组织中的表达水平。同时探讨DDB1表达与患者临床特征的相关性。样本筛选遵循严格标准：仅纳入TCGA病理确诊的癌症样本，排除正常组织及非原发肿瘤样本。数据按肿瘤分期和淋巴结转移状态进行分层处理，采用t检验评估。

### 1.3 Kaplan-Meier Plotter分析

Kaplan-Meier Plotter在线数据库是肿瘤预后分析平台。本研究通过数据分析DDB1在LUAD预后评估中的生物学意义。输入基因DDB1并癌症类型选择LUAD。生存终点设定为总生存期（overall survival, OS）和首次进展期（first progression, FP），分组采用数据库自动优化最佳截断值。样本纳入基因表达综合数据库（Gene Expression Omnibus, GEO）原发性LUAD病例，排除转移性肿瘤及随访缺失数据。生存差异通过Log-rank检验评估，HR及95%CI由Cox回归模型计算。

### 1.4 GEPIA（Gene Expression Profiling Interactive Analysis）分析

GEPIA平台支持基因表达差异分析、生存率分析等多项功能。研究通过该平台评估DDB1在LUAD中的表达。在"Expression DIY"模块输入DDB1基因，选择LUAD及GTEx正常肺组织作为对照。设定显著性阈值P<0.05，生成DDB1在肿瘤与正常组织中的箱线图。TCGA样本仅纳入病理确诊的LUAD原发肿瘤，正常组织样本被排除。GTEx对照组采用健康供者的肺组织数据。

### 1.5 GeneMANIA分析

使用GeneMANIA数据库对DDB1蛋白进行相互作用分析。该数据库主要功能是预测基因功能和构建基因相互作用网络。当输入DDB1基因后，系统会基于蛋白互作网络筛选相关基因。通过分析基因与DDB1的相互作用关系，可预测其参与的生物学功能。所有结果均通过GeneMANIA特有的算法模型进行计算输出。

### 1.6 功能富集

我们从GEPIA数据库的“Similar Genes”板块筛选出与DDB1在LUAD和癌旁组织中表达关联最密切的前100个基因，并将基因列表上传至Metascape平台。在物种选项选择人类后，执行GO生物过程富集分析。分析参数设定为P值阈值<0.05，蛋白质互作网络来源于STRING数据库，置信度阈值设为≥0.7。通过超几何检验进行显著性验证，同时应用伪发现率（false discovery rate, FDR）校正。最终保留FDR<0.1的显著通路，并剔除功能未注释的基因。网络核心节点的筛选标准为度中心性≥5，以此确定关键调控节点。

### 1.7 免疫细胞浸润分析

针对LUAD的免疫特征研究，我们通过TISIDB数据库开展分析。选定DDB1作为靶基因后，运用肿瘤免疫评估资源（Tumor Immune Estimation Resource, TIMER）算法评估该基因表达与免疫细胞浸润水平的相关性。我们计算了免疫细胞丰度值，并绘制出Spearman相关性热图。结果显示DDB1与淋巴细胞、免疫调节分子及趋化因子存在显著相关性。

### 1.8 TIMER分析

我们利用TIMER这一肿瘤分析工具开展研究。通过其内置的"Diff Exp"功能模块，可对DDB1基因进行差异表达检测。本研究基于TCGA公共数据库，系统评估了该基因在多种恶性肿瘤中的转录水平差异。

### 1.9 TNMplot分析

本研究使用TNMplot平台分析DDB1的表达差异。研究数据来源于配对的LUAD组织及癌旁正常组织。操作选择"TN-plot: compare Tumor and Normal"模块，输入DDB1基因。参数设置包括勾选"paired tumor and adjacent normal tissues"选项，并选定LUAD癌症类型。系统最终自动生成对比箱线图。

### 1.10 单细胞测序分析

基于scTIME平台的GSE131907数据集，首先通过聚类算法解析细胞亚型，继而探究DDB1在免疫细胞亚群内的分布特征。结合肿瘤微环境（tumor microenvironment, TME）单细胞数据库，系统性分析该基因在不同细胞类型中的表达差异。最终借助热图技术进行数据量化与图形化展示，对整体表达特征进行全面解析。

### 1.11 人类蛋白质表达图谱（Human Protein Atlas, HPA）分析

本研究利用公开的HPA数据库中的免疫组化数据，评估DDB1蛋白在LUAD组织与正常肺组织中的表达差异。

### 1.12 免疫组化

组织标本经过标准二甲苯脱蜡处理后，采用pH 6.0柠檬酸盐缓冲液进行抗原修复。实验体系中添加稀释比例为1:100的DDB1一抗，于4 ^o^C冰箱中孵育12 h。PBS漂洗3次，与辣根过氧化物酶标记二抗在室温下共育1 h，最后使用新鲜配置的DAB底物完成显色。经苏木精对比染色后，通过光学显微镜观察胞质内深蓝色颗粒分布情况。双盲评估方案由两位资深实验技术人员独立执行，评分系统包含双重维度：胞浆染色强度按四级标准（0-3分）判定，阳性细胞比例依五级标准（0-4分）统计。将强度评分与阳性率评分相乘获得总积分，以12分为界值进行分组。

### 1.13 统计学分析

数据分析采用SPSS 24.0软件完成，图形绘制使用GraphPad Prism 8软件实现。符合正态分布的定量数据采用均数±标准差进行描述，组间差异由独立样本t检验评估。离散型数据采用频数分布表示，组间差异检验选用卡方分析。生存曲线的单因素分析行Kaplan-Meier检验，统计学差异判定依赖Log-rank检验。单因素筛选显著的影响因子引入Cox多因素风险模型。显著性阈值取P<0.05。

## 2 结果

### 2.1 DDB1在LUAD中的高表达

为阐明DDB1的致癌生物学特性，通过TIMER平台进行生物信息学分析（图1A）。结果显示，DDB1 mRNA在LUAD、肺鳞状细胞癌、乳腺癌等肿瘤组织中表达显著高于正常组织（P<0.05）。通过UALCAN平台对LUAD样本进行解析发现相较于正常组织，肿瘤部位的DDB1 mRNA表达存在显著上调趋势（P<0.001）（图1B）。GEPIA平台验证显示，LUAD中DDB1 mRNA表达呈现相同趋势（P<0.01）（图1C、1D）。通过检测TNMplot数据库内259对LUAD与正常配对样本，发现DDB1在恶性肿瘤组织中呈现显著高表达（P<0.001）（图1E）。

**图1:**
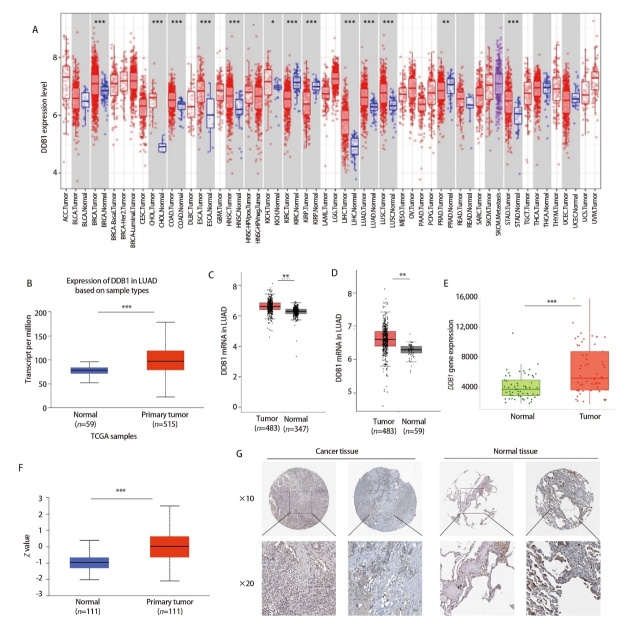
DDB1在LUAD中的表达上调。A：基于TIMER平台分析DDB1在不同肿瘤中的表达情况；B：UALCAN平台中（TCGA数据库）LUAD组织中DDB1 mRNA表达高于癌旁组织；C：GEPIA平台中（TCGA和GTEx数据库）DDB1 mRNA在肿瘤和正常组织中的表达；D：GEPIA平台中（TCGA数据库）DDB1 mRNA在肿瘤和正常组织中的表达；E：TNMplot平台中DDB1 mRNA在配对LUAD癌组织与癌旁组织中的表达情况；F：在UALCAN平台中（CPTAC数据库），DDB1蛋白在LUAD组织中高表达；G：在人类蛋白质图谱数据库中免疫组化检测DDB1在LUAD组织和癌旁肺组织中的蛋白表达。*P<0.05，**P<0.01，***P<0.001。

为进一步验证DDB1表达特征，我们通过CPTAC数据库检测蛋白水平（图1F）。在LUAD组织中，DDB1蛋白的表达量明显高于癌旁正常组织（P<0.001）。HPA平台的分析数据进一步支持这一发现，显示该蛋白在肿瘤中维持异常高表达状态（图1G）。

### 2.2 DDB1在LUAD患者中的表达与临床指标关联

本研究首先基于TCGA数据解析DDB1表达与临床参数的关联，发现该基因在不同临床分期（P<0.001）及不同淋巴结转移（P<0.001）中显著高表达（图2A、2B）。进一步采用Kaplan-Meier Plotter进行生存分析，结果显示DDB1高表达患者OS明显劣于低表达组（HR=2.76, 95%CI: 2.11-3.60, P<0.001）（图2C）。FP预后分析也验证了这一关联（HR=1.96, 95%CI: 1.51-2.54, P<0.001）（图2D）。综合多维度证据，DDB1被确认为LUAD的关键致癌因子，其作为诊断标志物和治疗靶点的双重功能值得深入探索。

**图2:**
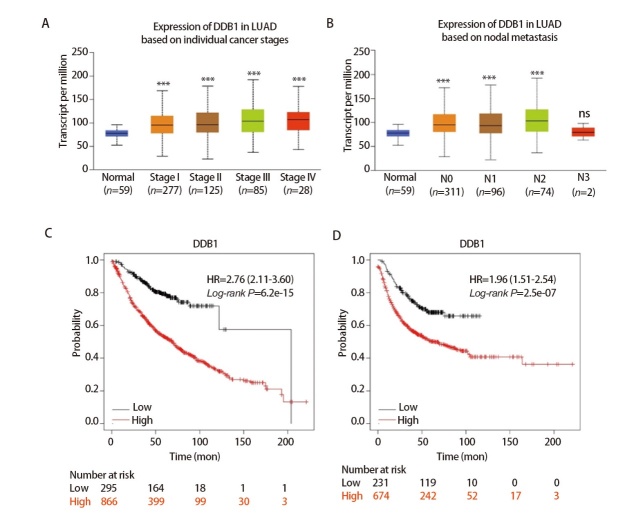
DDB1与LUAD的临床相关性。A、B：TCGA数据库（UCLCAN）中不同肿瘤分期（A）和淋巴结转移（B）的LUAD中DDB1的表达；C：基于Kaplan-Meier Plotter，根据DDB1在LUAD中的表达水平计算的OS的生存曲线；D：基于Kaplan-Meier Plotter，根据DDB1在LUAD中的表达水平计算的FP的生存曲线。***P<0.001。

### 2.3 DDB1的相互作用网络

我们采用GeneMANIA平台构建DDB1相互作用网络，以研究其结合蛋白。分析显示，DDB1与多个功能相关蛋白存在广泛相互作用（图3A）。图中可见DDB1直接与DDB2、XPC等蛋白互作，其互作对象还包括DCAF家族成员（如DCAF1、DCAF4等）及RAD23B蛋白。这些物理相互作用提示DDB1的核心功能依赖上述分子。DDB2与XPC的相互作用验证了DDB1在核苷酸切除修复（nucleotide excision repair, NER）系统中的关键功能。DCAF家族蛋白的富集表明DDB1可能参与形成CRL4泛素连接酶复合物，该复合物可能调控底物泛素化降解过程。网络类型分析显示，DDB1与EROC4、CPSF1等蛋白存在共表达或遗传互作，这暗示其在转录调控或RNA加工中具有功能协同性。上述结果为深入解析DDB1的功能提供了新线索。

**图3:**
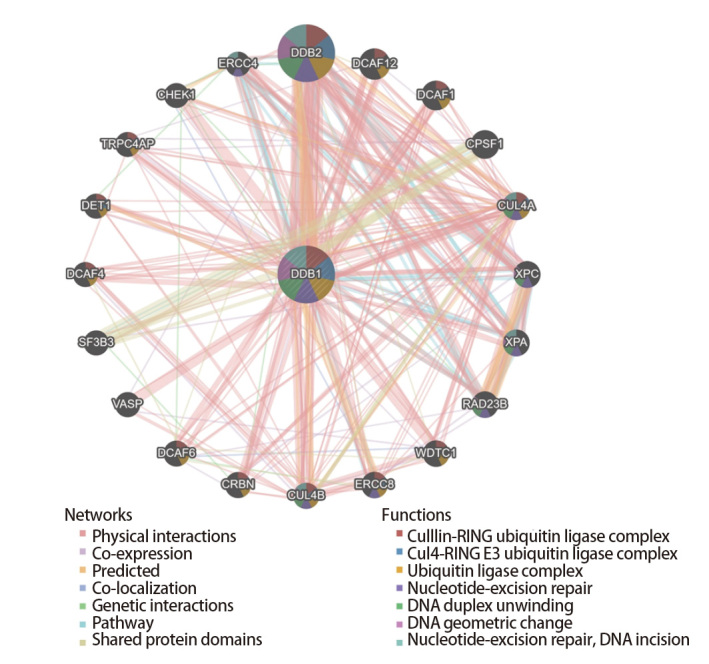
DDB1的相互作用蛋白和共表达基因

### 2.4 DDB1相关基因的功能富集

为研究DDB1的生物学功能，我们开展基因本体（Gene Ontology, GO）功能富集分析。首先通过GEPIA数据库调取TCGA中LUAD相关数据，筛选出与DDB1相关性最强的100个基因。随后使用Metascape平台对这些基因进行功能富集分析。结果显示，DDB1主要参与三大生物学过程（图4A、4B），包括“mRNA代谢过程”、“DNA代谢调控”和“染色质重塑”。这些发现揭示DDB1在基因表达调控中的多重作用。值得注意的是，“RNA 3'端加工”和“RNA核输出调控”的富集，提示DDB1可能参与转录后调控。该功能与其已知的泛素连接酶功能形成互补。在DNA损伤修复方面，DDB1相关基因显著富集于“UV响应”和“DNA生物合成调控”。此外，“核糖核蛋白复合体形成”与“蛋白质细胞器定位”的关联性，表明DDB1可能通过调控蛋白稳定性维持细胞器功能。

**图4:**
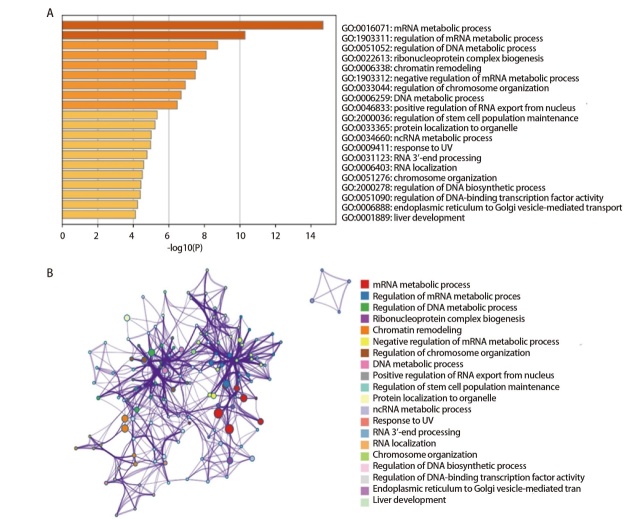
DDB1相关基因的GO功能富集。A：基于DDB1表达富集最相关途径，数据来源于Genemania平台；B：根据ID着色的附件及途径网络。图像由Metascape平台生成。

### 2.5 DDB1与免疫细胞浸润的关系

为探究DDB1与免疫细胞浸润的关系，我们通过TISIDB平台开展Spearman分析。结果显示，DDB1基因在LUAD中的表达与免疫调控分子呈显著负相关（图5A-5L）。例如，DDB1与CTLA4等免疫检查点基因呈现负相关，提示其高表达可能抑制免疫调控分子活性。这种抑制作用可能通过调控TME，最终影响机体免疫应答效率。

**图5:**
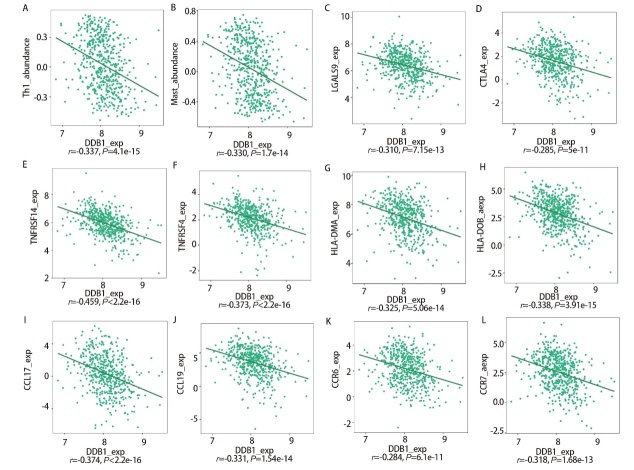
DDB1表达与免疫细胞浸润之间的关系。A：Th1（r=-0.337）；B：肥大细胞（r=-0.330）；C：LGALS9（r=-0.310）；D：CTLA4（r=-0.285）；E：TNFRSF14（r=-0.459）；F：TNFRSF4（r=-0.373）；G：HLA-DMA（r=-0.325）；H：HLA-DOB（r=-0.338）；I：CCL17（r=-0.374）；J：CCL19（r=-0.331）；K：CCR6（r=-0.284）；L：CCR7（r=-0.318）。

### 2.6 DDB1在LUAD中的单细胞分析

本研究整合了来自scTIME网络的单细胞转录组数据。图6A通过UMAP可视化展示40种免疫细胞的聚类分布，图6B则揭示DDB1在不同免疫细胞中的表达特征。为精准定位TME中DDB1的优势表达细胞，课题组实施了跨数据集比较分析。GSE117570数据集（包含4例NSCLC患者的11,453个细胞）分析结果表明，B淋巴细胞、T淋巴细胞及树突状细胞呈现显著的DDB1表达信号（图6C）。在另一EMTAB6149数据集（含5例患者的40,218个细胞）中，肺泡巨噬细胞与T/B淋巴细胞被确认为DDB1的主要表达载体（图6D）。

**图6:**
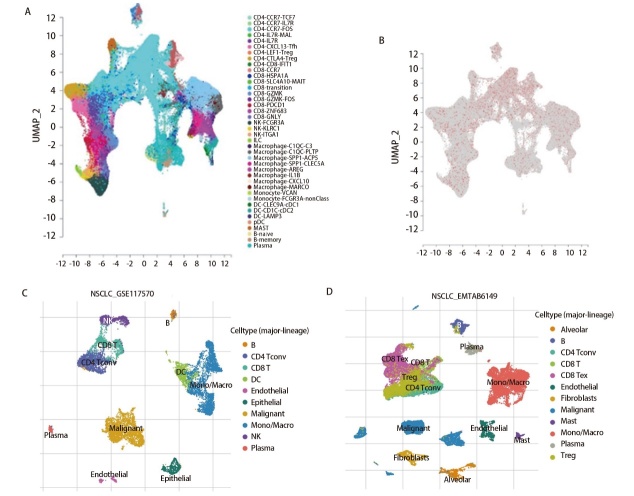
DDB1在LUAD中的单细胞分析。A：40种免疫细胞的DDB1富集特征；B：免疫浸润细胞DDB1表达谱系分析；C：GSE117570数据集10种细胞类型分布；D：EMTAB6149数据集12种细胞类型分布。

### 2.7 DDB1在组织芯片中高表达

基于免疫组化检测数据，LUAD组织中DDB1的阳性率较癌旁组织呈现显著升高（P<0.001）（图7A、7B），这一现象与HPA数据库记录具有一致性。生存分析显示，相较于高表达群体，DDB1低表达患者的OS明显延长（P<0.001）（图7C）。通过对比临床基线数据，不同DDB1表达水平的病例在特征分布上尚未观察到显著统计学差异（表1）。Cox回归分析证实DDB1具有独立预后价值（表2）。单因素分析显示，DDB1高表达（P<0.001）、临床分期（P=0.005）、T分期（P<0.001）、N分期（P=0.047）及肿瘤原发灶-淋巴结-转移（tumor-node-metastasis, TNM）分期（P=0.002）均为显著危险因素。多因素分析中，仅DDB1高表达（P<0.001）与T分期（P=0.007）保持独立预后意义。以上结果提示DDB1可作为LUAD预后预测指标。

**图7:**
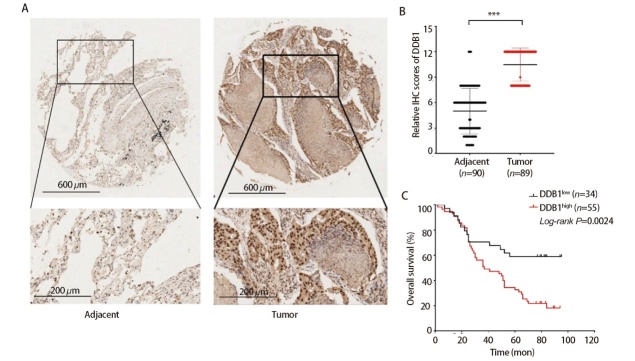
DDB1在LUAD组织中高表达及其与临床预后的关系。A：DDB1在LUAD组织芯片中癌组织高表达（右侧）及癌旁组织低表达（左侧）的代表性图片；B：LUAD组织芯片中DDB1在癌组织及癌旁组织的表达情况比较；C：DDB1高表达的LUAD患者OS短。***P<0.001。

**表1:** DDB1表达与LUAD患者临床病理参数的关系

Variable	Total (n=89)	DDB1 expression		P value
		Low (n=34)	High (n=55)	
Age (yr)				0.055
<65	57 (64.04%)	26 (76.47%)	31 (56.36%)	
≥65	32 (35.96%)	8 (23.53%)	24 (43.64%)	
Age (yr)				0.233
<60	40 (44.94%)	18 (52.94%)	22 (40.00%)	
≥60	49 (55.06%)	16 (47.06%)	33 (60.00%)	
Gender				0.467
Female	41 (46.07%)	14 (41.18%)	27 (49.09%)	
Male	48 (53.93%)	20 (58.82%)	28 (50.91%)	
Clinical stage				0.229
I-II	70 (78.65%)	29 (85.29%)	41 (74.55%)	
III	19 (21.35%)	5 (14.71%)	14 (25.45%)	
T stage				0.228
T1	50 (56.18%)	23 (67.65%)	27 (49.09%)	
T2	29 (32.58%)	8 (23.53%)	21 (38.18%)	
T3-T4	10 (11.24%)	3 (8.82%)	7 (12.73%)	
N stage				0.887
N0	55 (61.80%)	22 (64.71%)	33 (60.00%	
N1	19 (21.35%)	7 (20.59%)	12 (21.82%)	
N2-N3	15 (16.85%)	5 (14.71%)	10 (18.18%)	
TNM stage				0.345
I	43 (48.31%)	19 (55.88%)	24 (43.64%)	
II	26 (29.21%)	10 (29.41%)	16 (29.09%)	
III-IV	20 (22.47%)	5 (14.71%)	15 (27.27%)	
TNM: tumor-node-metastasis.				

**表2:** Cox单因素及多因素风险模型分析影响LUAD患者预后的危险因素

Variables	Univariate analysis			Multivariate analysis		
	HR	95%CI	P	HR	95%CI	P
DDB1 expression	3.906	2.245-6.798	<0.001	4.031	2.162-7.517	<0.001
Gender	1.130	0.674-1.893	0.644			
Clinical stage	2.294	1.278-4.117	0.005	1.080	0.550-2.120	0.822
Age (≥65 yr)	1.615	0.960-2.719	0.071			
T stage	2.222	1.542-3.202	<0.001	2.317	1.259-4.263	0.007
N stage	1.402	1.005-1.956	0.047	1.184	0.644-2.176	0.586
TNM stage	1.659	1.203-2.288	0.002	0.886	0.410-1.918	0.759

## 3 讨论

作为威胁人类健康的主要恶性肿瘤，肺癌的临床诊疗进展始终是医学界关注焦点，相关报告证实其致死率位居各类癌症前列^[12]^。虽然LUAD特异性生物标志物已有明确界定，但药物敏感性差异及现有分子标志的临床应用瓶颈，仍驱动着新型疗效预测模型与个体化治疗靶点的持续发掘。DDB1作为CUL4-DDB1泛素连接酶复合体的核心衔接蛋白参与调控DNA损伤修复、表观遗传修饰及细胞周期检查点激活等关键生物学过程。多项研究证实DDB1在卵巢癌^[13]^、乳腺癌^[14]^和结直肠癌^[15]^中存在明显过表达现象，揭示了该蛋白与肿瘤生物学行为及临床预后的密切相关性。现有研究对DDB1在LUAD中的功能尚未充分阐明。本研究综合运用生物信息学技术解析其表达谱特征，相关发现可为靶向药物研发奠定理论基础，助力精准医疗临床实践。

我们的研究发现，DDB1 mRNA的表达量显著高于正常组织，这一现象在不同癌种中普遍存在。本研究聚焦DDB1在LUAD中的特异性表达，通过多数据库联合分析发现，癌组织中的DDB1 mRNA和蛋白表达均明显上调，且高表达与不良预后有关。基于LUAD组织芯片的实验验证，进一步确认了DDB1的促肿瘤特性。值得注意的是，淋巴结转移作为癌症扩散的关键路径，直接影响治疗方案和患者生存率。本研究揭示DDB1表达水平与淋巴结转移状态存在显著关联，提示其可作为转移风险评估的新型生物标志物。

此外，我们挖掘了DDB1蛋白的候选互作蛋白，并富集其共表达基因及相关通路。部分蛋白质已被证实参与癌症发生，例如DCAF1（VprBP）作为非典型激酶，可下调肿瘤抑制基因转录，显著增加结肠癌与前列腺癌风险^[16]^。研究^[17]^显示CPSF1在替代选择性多聚腺苷酸化（alternative polyadenylation, APA）过程中发挥核心作用，其异常表达与多种恶性肿瘤的发生发展存在关联。由DDB1与CUL4构成的E3泛素连接酶系统具有多组织分布特征，能够通过精确调控细胞增殖周期、动态改变染色质构象以及修复基因组损伤等方式执行生物学功能^[18]^。针对此类蛋白互作网络的研究，为开发靶向DDB1的抑制剂或阻断其相互作用的新型治疗策略奠定了理论基础。

GO分析显示DDB1参与基因表达调控、DNA损伤修复及RNA转录后调控过程。有研究^[19]^表明DDB1通过影响核糖体DNA转录参与核糖体合成，驱动初始T细胞从静止态向代谢活跃态转变。但DDB1调控核糖体合成的具体机制仍需深入探索。基因组的完整性维护依赖于多元化的修复通路。损伤严重性取决于外界刺激的强度和作用时间，具体表现为碱基配对异常、氧化性甲基化、链间交联或双链断裂等多种形式。对应的修复策略包括错配校正、碱基切除、核苷酸切除以及同源定向重组等途径。这些系统的协同运作确保了遗传稳定性，其功能异常已被证实会导致基因组紊乱并促进癌变过程^[20]^。DDB1作为必需因子，通过与DDB2结合形成紫外线损伤识别复合体，进而召集核苷酸切除通路中的修复蛋白启动损伤修复^[21]^。

研究^[22]^证明，免疫成分的浸润程度是疾病演进和治疗敏感性的重要机制。生物信息学分析显示，DDB1的表达模式与Th1、肥大细胞等效应细胞的浸润深度存在密切相关性。这表明其可能通过调控免疫细胞功能参与肿瘤进程。研究^[23,24]^表明，肺癌发生与TME密切相关。TME由细胞外基质和基质细胞（包括免疫细胞）组成，其动态变化直接影响癌细胞的发生发展。单细胞测序分析显示，DDB1在TME的T细胞、肺泡巨噬细胞以及树突状细胞等细胞中高表达。这提示DDB1可能通过调控TME影响癌症进程。免疫细胞不仅影响患者预后，还参与免疫监视、逃逸及肿瘤演进等过程。不同免疫细胞对肿瘤存活和耐药性产生差异影响。例如，肿瘤相关巨噬细胞可释放细胞因子，抑制效应免疫细胞。其还会募集免疫抑制细胞至TME^[25]^，导致患者预后不良。而CD8^+^ T细胞能有效杀伤浸润癌细胞，改善临床结局^[26]^。对晚期肺癌患者，免疫疗法兼具疗效与安全性优势。PD-L1是应用最广的免疫检查点抑制剂，对NSCLC、黑色素瘤等疗效显著。CTLA4抑制剂则可提升癌症免疫治疗效果^[27]^。本研究发现在LUAD中DDB1与CTLA4存在相关性，这提示DDB1或可成为LUAD免疫治疗的新靶点。虽然DDB1与免疫浸润相关，但其作为治疗标志物的价值仍需深入验证。

本研究采用生物信息学分析方法，结合组织芯片，系统解析了DDB1在LUAD肿瘤组织中的表达规律，揭示其在疾病转归预测和免疫治疗敏感性评估中的重要生物学意义。然而研究存在局限性，生物信息学工具间存在结果差异，主要源于算法差异和数据格式多样性。软件更新频率和参数设置不同加剧了验证难度，组织芯片验证可能存在选择偏倚。当前标本量有限，后续需扩大样本验证结论。未来研究将开展功能实验，验证DDB1对肿瘤进展的具体影响。进一步构建LUAD小鼠模型，观察DDB1表达调控与肿瘤发展的关系。虽然DDB1有望成为治疗靶点，仍需功能实验验证其机制并探索治疗策略。本研究系统探讨了DDB1的表达特征，为理解其在LUAD等肿瘤中的作用奠定基础。

## References

[1] Chang GC, Chiu CH, Yu CJ, et al. Low-dose CT screening among never-smokers with or without a family history of lung cancer in Taiwan: a prospective cohort study. Lancet Respir Med, 2024, 12(2): 141-152. doi: 10.1016/S2213-2600(23)00338-7 38042167

[2] Liang J, Bi G, Huang Y, et al. MAFF confers vulnerability to cisplatin-based and ionizing radiation treatments by modulating ferroptosis and cell cycle progression in lung adenocarcinoma. Drug Resist Updat, 2024, 73: 101057. doi: 10.1016/j.drup.2024.101057 38266355

[3] Seguin L, Durandy M, Feral CC. Lung adenocarcinoma tumor origin: A guide for personalized medicine. Cancers, 2022, 14(7): 1759. doi: 10.3390/cancers14071759 PMC8996976 35406531

[4] Xiao Y, Liu P, Wei J, et al. Recent progress in targeted therapy for non-small cell lung cancer. Front Pharmacol, 2023, 14: 1125547. doi: 10.3389/fphar.2023.1125547 36909198 PMC9994183

[5] Chhouri H, Alexandre D, Grumolato L. Mechanisms of acquired resistance and tolerance to EGFR targeted therapy in non-small cell lung cancer. Cancers (Basel), 2023, 15(2): 504. doi: 10.3390/cancers15020504 PMC9856371 36672453

[6] Hu H, Cheng R, Wang Y, et al. Oncogenic KRAS signaling drives evasion of innate immune surveillance in lung adenocarcinoma by activating CD47. J Clin Invest, 2023, 133(2): e153470. doi: 10.1172/JCI153470 PMC9843062 36413402

[7] Bi YY, Chen Q, Yang MY, et al. Nanoparticles targeting mutant p 53 overcome chemoresistance and tumor recurrence in non-small cell lung cancer. Nat Commun, 2024, 15(1): 2759. doi: 10.1038/s41467-024-47080-3 PMC10980692 38553451

[8] Raisch J, Dubois ML, Groleau M, et al. Pulse-SILAC and interactomics reveal distinct DDB1-CUL4-associated factors, cellular functions, and protein substrates. Mol Cell Proteomics, 2023, 22(10): 100644. doi: 10.1016/j.mcpro.2023.100644 PMC10565876 37689310

[9] Lim HE, Lim HJ, Yoo HY. Interaction of DDB1 with NBS1 in a DNA damage checkpoint pathway. Int J Mol Sci, 2024, 25(23): 13097. doi: 10.3390/ijms252313097 PMC11642328 39684807

[10] Shan Y, Mao B, Jin Y, et al. Expression of DDB1 is associated with subtypes of epithelial ovarian cancer and predicts clinical outcomes. Tissue Cell, 2023, 82: 102072. doi: 10.1016/j.tice.2023.102072 36934683

[11] Yu J, Ge S. PRPF 19 functions in DNA damage repair and gemcitabine sensitivity via regulating DDB1 in bladder cancer cells. Cytotechnology, 2024, 76(1): 85-96. doi: 10.1007/s10616-023-00599-7 38304628 PMC10828380

[12] Huang J, Deng Y, Tin MS, et al. Distribution, risk factors, and temporal trends for lung cancer incidence and mortality: a global analysis. Chest, 2022, 161(4): 1101-1111. doi: 10.1016/j.chest.2021.12.655 35026300

[13] Tang ZY, Wang XM, Xu CW, et al. DCAF 13 promotes ovarian cancer progression by activating FRAS1-mediated FAK signaling pathway. Cell Mol Life Sci, 2024, 81(1): 421. doi: 10.1007/s00018-024-05446-2 PMC11455852 39367995

[14] Wang M, Fu L, Tian J, et al. Function and prognosis analysis of nucleolus protein DCAF 13 in breast cancer. Transl Cancer Res, 2023, 12(12): 3744-3751. doi: 10.21037/tcr-23-1923 38197079 PMC10774066

[15] Luo D, Chen M, Li Q, et al. CUL4B-DDB1-COP1-mediated UTX downregulation promotes colorectal cancer progression. Exp Hematol Oncol, 2023, 12(1): 77. doi: 10.1186/s40164-023-00440-z PMC10483726 37679762

[16] Shin Y, Kim S, Liang G, et al. DCAF1/VprBP triggers melanomagenic gene silencing through histone H2A phosphorylation. Biomedicines, 2023, 11(9): 2552. doi: 10.3390/biomedicines11092552 PMC10526264 37760992

[17] Xudong X, Heng L, Benchao C, et al. Integrated RNA expression and alternative polyadenylation analysis identified CPSF1-CCDC 137 oncogenic axis in lung adenocarcinoma. Environ Toxicol, 2024, 39(4): 2405-2416. doi: 10.1002/tox.24105 38174951

[18] Tseng C, Han Y, Lv Z, et al. The CRL4^DCAF6^ E 3 ligase ubiquitinates CtBP1/2 to induce apoptotic signalling and promote intervertebral disc degeneration. J Mol Med (Berl), 2023, 101(1-2): 171-181. doi: 10.1007/s00109-022-02277-1 36688959

[19] Sharma M, Shaw AS. Nucleolar condensates: A cellular machinery necessary for T cell activation. J Cell Biol, 2023, 222(10): e202309067. doi: 10.1083/jcb.202309067 PMC10513034 37733425

[20] Prabhu KS, Kuttikrishnan S, Ahmad N, et al. H2AX: A key player in DNA damage response and a promising target for cancer therapy. Biomed Pharmacother, 2024, 175: 116663. doi: 10.1016/j.biopha.2024.116663 38688170

[21] Jang S, Raja SJ, Roginskaya V, et al. UV-DDB stimulates the activity of SMUG 1 during base excision repair of 5-hydroxymethyl-2’-deoxyuridine moieties. Nucleic Acids Res, 2023, 51(10): 4881-4898. doi: 10.1093/nar/gkad206 36971122 PMC10250209

[22] Lu Q, Kou D, Lou S, et al. Nanoparticles in tumor microenvironment remodeling and cancer immunotherapy. J Hematol Oncol, 2024, 17(1): 16. doi: 10.1186/s13045-024-01535-8 PMC10986145 38566199

[23] Kraemer AI, Chong C, Huber F, et al. The immunopeptidome landscape associated with T cell infiltration, inflammation and immune editing in lung cancer. Nat Cancer, 2023, 4(5): 608-628. doi: 10.1038/s43018-023-00548-5 37127787 PMC10212769

[24] Jayaram MA, Phillips JJ. Role of the microenvironment in glioma pathogenesis. Ann Rev Pathol, 2024, 19: 181-201. doi: 10.1146/annurev-pathmechdis-051122-110348 37832944

[25] Huang R, Kang T, Chen S. The role of tumor-associated macrophages in tumor immune evasion. J Cancer Res Clin Oncol, 2024, 150(5): 238. doi: 10.1007/s00432-024-05777-4 PMC11076352 38713256

[26] Jacobs C, Shah S, Lu WC, et al. HSF 1 inhibits antitumor immune activity in breast cancer by suppressing CCL5 to block CD8^+^ T-cell recruitment. Cancer Res, 2024, 84(2): 276-290. doi: 10.1158/0008-5472.CAN-23-0902 37890164 PMC10790131

[27] Sposito M, Eccher S, Scaglione I, et al. The frontier of neoadjuvant therapy in non-small cell lung cancer beyond PD-(L) 1 agents. Expert Opin Biol Ther, 2024, 24(10): 1025-1037. doi: 10.1080/14712598.2024.2408292 39311630

